# The archaeology of climate change: a blueprint for integrating environmental and cultural systems

**DOI:** 10.1038/s41467-025-60450-9

**Published:** 2025-06-13

**Authors:** Ariane Burke, Matt Grove, Andreas Maier, Colin Wren, Michelle Drapeau, Timothée Poisot, Olivier Moine, Solène Boisard, Laurent Bruxelles

**Affiliations:** 1 Département d’Anthropologie, Université de Montréal, Montréal, QC Canada; 2 Archaeology, Classics and Egyptology, University of Liverpool, Liverpool, UK; 3 Institute for Prehistoric Archaeology, University of Cologne, Cologne, Germany; 4 Anthropology Department, University of Colorado, Colorado Springs, CO USA; 5 Département de Sciences Biologiques, Université de Montréal, Montréal, QC Canada; 6 Laboratoire de Géographie Physique: Environnements quaternaires et actuels. UMR 8591 CNRS- Paris 1-UPEC, Thiais, France; 7 TRACES, UMR 5608, CNRS, Toulouse, France & GAES, University of the Witwatersrand, Johannesburg, South Africa

**Keywords:** Climate change, Climate-change adaptation

## Abstract

Cultural systems play an important role in shaping the interactions between humans and the environment, and are in turn shaped by these interactions. However, at present, cultural systems are poorly integrated into the models used by climate scientists to study the interaction of natural and anthropogenic processes (i.e. Earth systems models) due to pragmatic and conceptual barriers. In this Perspective, we demonstrate how the archaeology of climate change, an interdisciplinary field that uses the archaeological record to explore human-environment interactions, is uniquely placed to overcome these barriers. We use concepts drawn from climate science and evolutionary anthropology to show how complex systems modeling that focuses on the spatial structure of the environment and its impact on demographic variables, social networks and cultural evolution, can bridge the gap between large-scale climate processes and local-scale social processes. The result is a blueprint for the design of integrative models that produce testable hypotheses about the impact of climate change on human systems.

## Abstract (web summary)

Here, the authors propose how climate change archaeology can explain how cultural systems impact interactions between humans and the environment, and vice versa, presenting a framework based in complex systems modelling.

## Introduction

The archaeology of climate change is an interdisciplinary field that explores the relationship between humans and their environment^1,2^. The archaeological record is a source of richly contextualised information about past human societies that provides a means of calibrating climate models, tracking human impacts on the environment and generating narratives in support of contemporary efforts to adapt to climate change^3^. Furthermore, archaeology provides a wealth of case studies with which to generate novel hypotheses that can be quantitatively tested^4^. Given the resurgence of interest in climate change research, bridging the gap between climate science and archaeology has become a priority (e.g. refs. ^3,5^). In this Perspective, we examine the different theoretical frameworks used to study biophysical, biological and cultural systems in hominin species with well documented cultural transmission. We identify bridging concepts that integrate these frameworks into a single modelling pipeline capable of generating testable hypotheses about human-climate interactions at both macro and microevolutionary scales.

Historically, climate scientists have focussed on discovering and explaining apparent correlations between the timing of significant thresholds in human evolution and climate events^6,7^. Correlative approaches have yielded numerous hypotheses about the impact of climate events on patterns of hominin speciation and dispersal (Box 1). Establishing causal links between climate events and changes in the palaeontological and archaeological records remains a major challenge, however^8–10^. In many of the studies cited in Box 1, the synchroneity of climate events and evolutionary change is assumed to indicate causality^7^ and adaptation and genetic drift are inferred mechanisms. But the causal pathways between climate change and culturally significant events such as the domestication of plants and animals^11,12^ and the transition to farming^13^, for example, are not always clear. This hinders our ability understand how climate change acts on human systems and highlights the need for a more integrated, interdisciplinary approach to climate science. Climate scientists adopt a range of theoretical frameworks, however, some of which may appear to be unreconcilable. Building conceptual bridges between these frameworks is a requirement if climate science is to advance^14,15^.

In this Perspective, we break down the conceptual barriers between existing theoretical frameworks to create an integrative workflow for the study of human-climate interactions. We use a modelling approach, grounded in complex systems theory, and highlight the role of spatial context—defined here as the abundance, spatial distribution, and connectivity of environmental variables relevant to human populations—as the critical link between biophysical and cultural systems. We begin by reviewing key theoretical frameworks in climate science, highlighting where conceptual and disciplinary boundaries exist. We then show how ecological models (see: Species Distribution Models), used in conjunction with Cumulative Cultural Evolution (CCE) theory, bridge the gap between evolutionary and anthropological approaches. A key aspect of CCE is its demonstration that patterns of cultural transmission and innovation are affected by the structure of the environment, producing evolutionary change in human culture (‘all that individuals learn from others that endures to generate customs and traditions’^16^ p. 938). This leads us to make a concrete proposal (Building Integrative Models) for the design of complex models that reconcile existing theoretical frameworks, exploit available datasets from the Earth and Social sciences, do not require extensive computing resources and can be adapted for use in different cultural and spatiotemporal contexts to make predictions about human/climate interactions that are testable using the archaeological record and Earth archives.

Box 1 Climate correlations Correlative approaches have yielded numerous hypotheses about the impact of climate events on patterns of hominin speciation, dispersal and cultural evolution (see figure below). The first evidence for stone tool manufacture coincides with the mid-Piacenzian Warm Period roughly 3.3 Ma^99^. *Homo erectus* emerges in the African fossil record and subsequently disperses out of Africa around 1.9 Ma and Acheulean stone tools appear ~1.75 Ma, coinciding with a transition to wetter conditions and the intensification of the Walker Circulation in East Africa^100^. The Eurasian expansion of the Acheulean coincides with the Mid-Pleistocene Transition from long (100 kyr) to short-term (40 kyr) glacial cycles^101^. The transition to the MSA and the emergence of *Homo sapiens* is synchronous with a major hydrological shift in Africa^102^. Neanderthals and Denisovans become extinct and *H. sapiens* disperses widely in Eurasia ~47 ka, at the end of an abrupt cold event (H5) prior to the onset of an interstadial (GI-12)^103^. The beginning of the Holocene ~12 ka coincides with the first signs of the domestication of plants and animals^104^, and a sudden cooling event at 8.2 ka coincides with the spread of the Neolithic^105^.

The impact of climate variability and abrupt climate change events on patterns of hominin adaptation have also been explored. For example, Vrba’s^106^ foundational work on ‘turnover pulses’ – periods characterised by significant temporal clustering of both extinction and speciation events – has been applied to the hominin record by White^107^ and Grove^108^ although the limited number of hominin species makes firm conclusions difficult. Potts’ ‘variability selection hypothesis’^109,110^ suggests that periods of high climatic variability rather than directional changes coincide with many major events in hominin evolution (e.g. ref. ^111^), including the origins of the Middle Stone Age and the emergence of *H. sapiens* in Africa^102^. Orbitally induced climate pulses have been identified as potential drivers of early hominin evolution in Africa through their impact on the physical environment, notably the aridification of eastern Africa^112,113^. Building on the work of Potts and colleagues, Maslin and colleagues^100^ argue that the unique geological and climatic conditions in eastern Africa, resulting in the presence of a series of ephemeral deep-water lakes in the region, produced precession-driven pulses of environmental variability that coincide with major events in the hominin record such as the first appearance of *Homo erectus* around 1.9 Ma. Hominin dispersals have also been linked to orbitally driven climate cycles, transforming landscapes and altering the location of biogeographic corridors, from the earliest dispersals out of Africa to the Late Pleistocene^38,114–117^.

**Figure:**
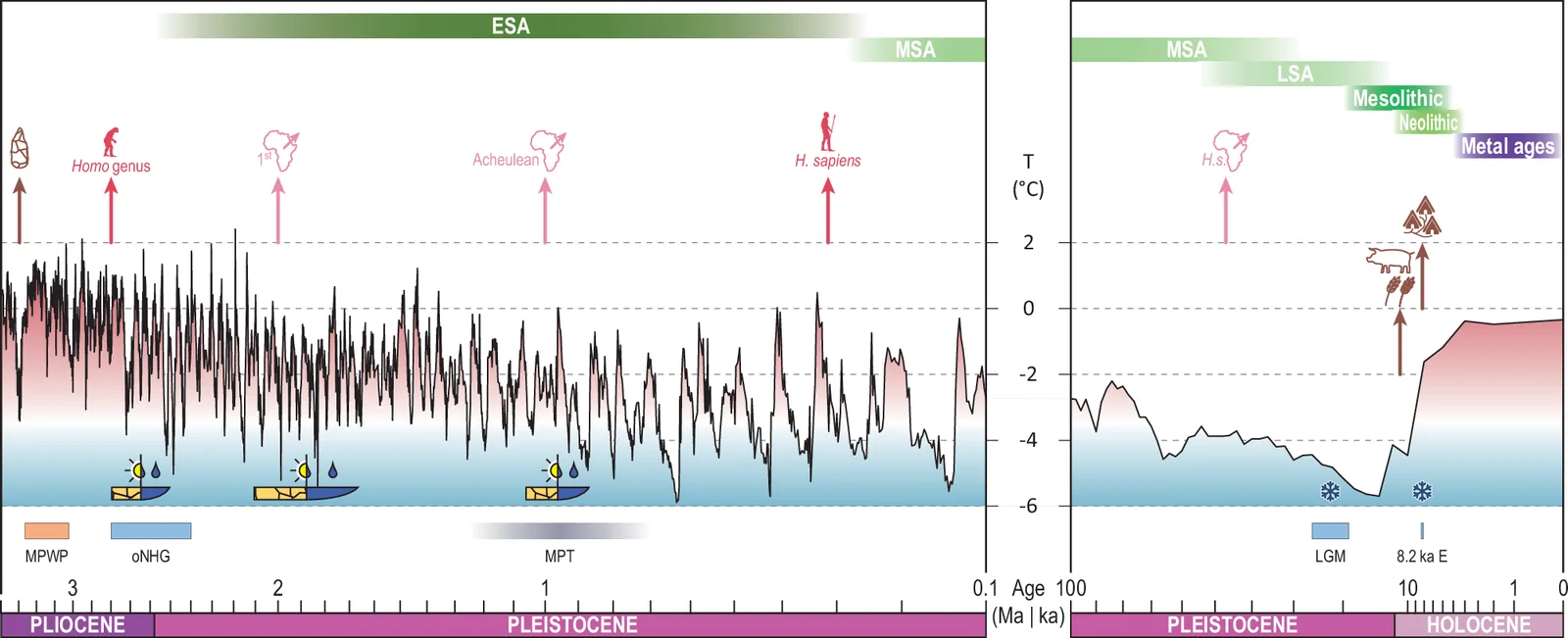

The figure above shows a generic timeline of key evolutionary events, including climate and bio-cultural data. African chrono-cultural periods, rather than their European equivalents, are illustrated because the African record is the longest and for brevity. Climate curve modified from ref. ^24^. MPWP = Mid-Piacenzian Warm Period, Sun/droplet = major transitions in cycles of aridification/lake extension in East Africa. ONHG = Onset of Northern Hemisphere Glaciation, MPT = Mid-Pleistocene Transition, H = onset of the Holocene, Snowflake: cold events including the LGM and the 8.2 ka event.

## Theoretical frameworks

Systems Theory is a useful, cross-disciplinary framework for modelling the complexities of climate/human interactions, but conceptually different approaches have been adopted within this framework. From an evolutionary perspective (see: Evolutionary Theory) adaptation is identified as the causal mechanism linking climate change to transformations in human systems and models tend to focus on macroevolutionary change. Models developed from an anthropological point of view (see: Anthropological Theory), on the other hand, focus on mechanisms of change arising from within human systems and are more attuned to microevolutionary processes. Although these approaches emphasise different causal mechanisms and scales of interaction, they are not incompatible. The difficulty lies in identifying bridging concepts that enable the design of complex, multiscalar models that integrate environmental and cultural systems.

### Systems theory

Complex systems theory is the dominant framework in climate change research today. A construct of physics, biology and the social sciences^17^, it highlights the importance of interdisciplinarity, the emergent properties of complex systems and the importance of local interactions as drivers of global processes, concepts that resonate with climate change archaeology^5,14^. Earth Systems Science (ESS), a variant of systems theory widely adopted by climate modellers^18^ encourages the integration of human variables in climate change models^19^, something human vulnerability and mitigation studies (e.g. ref. ^20,21^) that now routinely include economic and demographic variables attempt. Complex Adaptive Systems (CAS), a counterpart to ESS, is more widely adopted in the social and natural sciences^22,23^. From a CAS perspective, culture is a dynamic, complex system that interacts with the biosphere and other biological systems^22,24,25^. This definition of culture is compatible with evolutionary theory, but it also raises questions about the extent to which the cultural system evolves in a manner analogous to the genetic system (Box 2). Although complex systems theory emphasizes interdisciplinary and multiscalar approaches, different theoretical frameworks have been used to develop models that, as a result, fail to integrate the full range of complexity that a true complex system model requires.

Box 2 Cultural evolution Culture is not genetically transmitted, unlike the innate capacity for acquiring knowledge and skills by observation and inference on which it is based. Culture provides a second inheritance system (e.g. refs. ^118–120^) with three modes of transmission: ‘vertical’ (from parents to offspring, and vice versa), ‘oblique’ (from non-parental elders) and ‘horizontal’ (from peers). This generates new cultural traits that are not simply incremental advances over previous ones, and leads to the blending of multiple, previously separate ‘cultural lineages’ (i.e. traditions) via a reticulate process^121^.

The generation of cultural variability and subsequent selection can arise in a manner analogous to biological systems. For example, drift has been used to explain adaptively neutral, ‘stylistic’ cultural change^122^ and the loss of cultural variation in small populations (e.g. ref. ^123^). However, there are differences: while the genetic system relies on stochastic processes such as mutation and recombination to generate variability, cultural variation can be ‘guided’^124^. Furthermore, unlike natural selection, cultural selection can be deliberately undertaken to achieve a particular goal (e.g. ref. ^125^) and the progenitor selected for copying need not be the ‘fittest’ in any traditional Darwinian sense. Cultural transmission, for example, can also be biased by social learning strategies such as pay-off biased and conformist social learning^126,127^. Pay-off biased transmission is the only social learning strategy that can be considered truly Darwinian^128^. Cultural norms can emerge arbitrarily, and conformity leads to the loss of innovation irrespective of adaptive potential, although the interaction of natural and cultural selection can reinforce norms with genuine adaptive value^129^. Though empirical evidence for conformist bias is weak^130^ (but see ref. ^131^), there remains a conviction that conformity is a powerful force in human social learning and, since it operates intra- and inter-generationally, it is an example of cultural selection operating on both macroevolutionary and microevolutionary levels. Finally, cultural traits are transmitted rapidly relative to genetic transmission which allows hominins to adapt rapidly to changing environments while retaining the benefits of a ‘slower’ life-history (e.g. ref. ^132^).

### Evolutionary theory

Evolutionary theory integrates biophysical and biological systems and has obvious applications for climate change research, although it oftentimes lacks a clear articulation of the mechanisms that explain how microevolutionary change ends up shaping the macroevolutionary process. At its core, evolution implies the existence of (or potential for) adaptation. As a state, adaptation implies the possession of traits and behaviours that contribute to positive fitness; for example, heightened oxygen metabolism is a metabolic adaptation to high-altitude conditions in many mammals. As a process (a population undergoes adaptation in a given environment), adaptation is the family of mechanisms through which an adaptive state is reached, as the etymology (‘towards being fit’) suggests. Since Ernst Mayr’s work on speciation, the process of adaptation has been understood as the sequential, iterative application of the generation of diversity (through mutation, recombination, immigration, and gene flow) and the removal of some of this variation through selection.

In order to reframe evolutionary theory in the context of cultural evolution, it is important to generate an inventory of the mechanisms that drive evolutionary change. Classifying these mechanisms by whether they generate (mutation, recombination, gene flow) or filter (selection, drift) variability is a first step towards identifying analogues within the realm of cultural evolution. It is also important to consider whether these mechanisms operate at both microevolutionary and macroevolutionary scales. Even though macroevolution (which generates diversity above the species level) proceeds from microevolution (which occurs at the population level within species), there is no clear definition of the mechanisms and processes linking the two.

Cultural evolutionary theory applies evolutionary theory to the study of human systems (Box 2) on the grounds that culture, and the behavioural plasticity it provides, is a fitness-enhancing adaptation subject to natural selection (e.g. ref. ^26^). It has been argued, however, that cultural evolution is so fundamentally different from biological evolution that it cannot be meaningfully addressed within a Darwinian evolutionary framework^27,28^.

Most notably, differences between biological and cultural evolution, such as the mode of selection (Box 2), cast doubt on the primacy of external variables such as climate as drivers of cultural change. Whereas natural selection is a clearly defined mechanism that describes how environmental change drives the evolution of biological organisms, the cultural context in which cultural evolution occurs  produces competing demands on the process of selection that are not fully accounted for in the modern Darwinian synthesis. Systems theory provides the framework within which these competing demands can be reconciled, provided we acknowledge that human systems are open, multiscalar systems influenced by both internal contexts and external influences (e.g. ref. ^25^).

The Extended Evolutionary Synthesis (ESS) takes a different approach, bridging the gap between biological and cultural systems by highlighting mutual feedback relationships between natural and cultural selection (e.g. ref. ^29^). Niche construction emphasises that non-random cultural changes affect the impact of environmental conditions on individuals and societies and may thereby drive biological evolution by changing the context of ‘natural’ selection^30^. However, while ESS accounts for some aspects of cultural change within evolutionary theory, broadly speaking it has not addressed the accumulative nature of material culture production in human societies^31^, nor does it provide clear explanatory mechanisms^32,33^. Models produced from this perspective, therefore, will still fall short of being fully integrated.

### Anthropological theory

In Anthropology and its sister discipline Archaeology, ontological thinking competes with ecological approaches. Twentieth-century postmodernists rejected evolutionary theory as a suitable framework for the study of cultural variation and social transformation, the implication being that natural and human systems should be studied in isolation. Under the influence of thinkers such as Gibson^34^ and Bateson^35^, however, a more holistic view of human beings as both biological and social organisms has emerged as an alternative to the Cartesian dualism that previously dominated Western thought. From this perspective, cultural variation is the result of individual agency and the complex web of relations, organic and inorganic, within which agents are situated^36^. This approach is compatible with systems theory but repositions the study of material culture at the centre of the archaeology of climate change, highlighting both the necessity and the challenge of reconciling two very different, but potentially complementary, theoretical approaches.

### The archaeology of climate change

The archaeology of climate change harnesses the analytical power of species distribution models (SDMs) to study the impact of past climate events on the spatial behaviour of human populations (e.g. refs. ^37–45^). Much of this research focusses on the impact of biophysical processes on the spatial distribution of human populations and patterns of dispersal, inferred to be causally linked by adaptation. Cultural change is typically addressed after the modelling process is complete, using inferential arguments to draw links between climate shifts and chronologically correlated cultural patterns^8^. This is partly a result of a tendency for environmental archaeologists and human ecologists interested in macroevolutionary processes to gravitate towards environmental data and ecological models. Among the latter, the proponents of cumulative cultural evolution (CCE) adopt a demographic focus, examining the impact of population structure (demography) on rates and patterns of cultural change. Archaeologists interested in microevolutionary processes at local or regional scales, on the other hand, tend to adopt a more qualitative, historical approach to the study of human-environment interactions.

We argue, as others have before us^15^, that by adopting a unified approach and bridging the gaps between environmentally based, demographic and historical approaches, the archaeology of climate change can solve what is currently a major stumbling block in climate science: the design of complex models that integrate Earth and human systems effectively and can be used to study their interactions at micro and macroevolutionary scales, producing hypotheses that can be tested using the archaeological record and Earth archives. This is made possible by integrating CCE models and SDMs.

### Cumulative culture models

Cumulative cultural evolution (CCE), a concept that flows from cultural evolution theory (Box 2), emphasises the accumulation of variation over time, often described in terms of an increase in the complexity of material culture^46^. For example, what started as simple stone flaking accumulated changes over generations resulting in the development of compound projectile weapons with long production sequences combining many materials. Cumulative culture may be more broadly construed in terms of an increasing richness of cultural traits within a population, i.e. more types of tools or greater stylistic variability^47,48^. Put simply, CCE can be equated with the ‘standing on the shoulders of giants’ metaphor for scientific progress; the incremental advances made by any given scientist are made possible only by the wealth of scholarship that has gone before. Similarly, cultural evolution occurs only when culturally inherited traits undergo incremental improvement before being transmitted to future users, who can themselves add further innovation. While it is not uniquely human (e.g. refs. ^49,50^), cumulative culture is a key adaptation of our lineage^51^.

The CCE literature is extensive but it tends to focus on the internal (i.e., social and cognitive) mechanisms of innovation, transmission, and selection at the expense of operationalized measures that reflect alterations to artefact form or assemblage variability (but see in refs. ^52,53^). More promising for our purposes is its focus on population density, mobility, and connectivity, as these spatial parameters can be directly conditioned by climatic and environmental changes. Larger populations are expected to produce more innovation per unit time (since per capita innovation rate is viewed as a constant), and both formal models and simulations demonstrate that greater population density and more mobility increase rates of transmission, since both lead to higher encounter rates between individuals or groups^54,55^. Connectivity also regulates rates of cultural evolution since large ‘culturally panmictic’ populations may generate more variation per unit time, but they may also suppress new innovations^54,56^. Structured or ‘partially connected’ populations, by contrast, maintain both cultural diversity and a sufficiently large overall population to sustain high innovation rates. The cultural trajectories of sub-populations partially isolated by the fragmentation of areas of suitable habitat following climatic and environmental change may diverge, with periodic contact between them facilitating the spread and combination of any beneficial variants that arise in individual demes (see also^56–58^). The archaeological record reveals spatially explicit patterns of cultural production^59^ confirming a link between climate change, the structure of the environment and patterns of cultural transmission. Models of cultural evolution are rarely spatially explicit, however (e.g. ref. ^60^) hindering their integration with palaeocological and archaeological models.

### Species distribution models

In contrast to models of cultural evolution, Species Distribution Models (SDMs) use spatially explicit environmental variables to estimate the distribution and size of habitable ‘patches’ (see below). The resulting models can then be used to predict chronological trends in population size and connectivity, offering the possibility of measuring the impact of climate change. SDMs are well placed, therefore, to bridge the gap between models of cultural evolution and climate change.

Since their introduction in 1984^61^, ecology has seen a constant increase in the use of SDMs (also known as habitat suitability models, or ecological niche models), of which the most frequently used are presence-only models like Maximum Entropy, or more flexible presence-absence models such as Generalised Linear Models (GLM) and tree-based methods (including Random Forest and Boosted Regression Trees)^62^. The typical SDM pipeline begins with the selection and preparation of observation data and environmental variables in a geographic information system to produce a feature class (observations) and a stack of spatially continuous coverages describing a set of environmental variables (predictors). The distribution of values in the environmental variables at locations where presences are recorded is used to characterise ‘suitable’ environmental conditions relative to background locations. Depending on the specific model used to predict habitat suitability, these background locations can be drawn at random or based on the distance to known observations^63^. The resulting models can be projected onto geographical space, mapping the distribution of suitable habitat on a scale of 0 to 1 (1 being the optimal set of conditions), which can be further thresholded to reflect discrete categories of habitat suitability^64^. SDMs can be combined into ensemble models^65^, although simulation studies highlight that this does not always improve model quality^66^.

Global climate models typically operate at spatial resolutions of ~150 km, which is suitable for tracking macroevolutionary processes over large spatial domains. A range of statistical and machine learning techniques is available for increasing the spatial resolution of global climate models to scales more suitable for tracking microevolutionary processes^67^, including short-term patterns of mobility and subsistence. Finally, SDMs describe the relationship between species distributions and the physical environment, but whether they describe a species’ fundamental niche, its realised niche, or only some niche components is debatable^68^ and depends very much on how the model is specified and whether functionally relevant environmental variables were selected.

Archaeological applications use archaeological sites as records of human presence (observations) and paleoclimate variables (e.g. temperature, precipitation) in conjunction with other abiotic variables (e.g. topographic variables) as the predictors. SDMs require ecological variables with high spatial coverage, which means that climate models, rather than reconstructions based on climate proxies such as pollen counts, are the most suitable source of climate data. Sources of simulated paleoclimate data are increasingly available for past timeframes^69–73^.

One of the dangers of a modelling approach is misspecification, i.e., either ignoring data that can’t readily be quantified or using data based on availability^74^. Many, if not most, archaeological SDMs use a restricted set of variables, ignoring biotic factors such as the presence of competitors, for example. SDMs also assume an equilibrium state which may or may not be appropriate, although rather than increasing the error in the model, non-equilibrium presences tend to provide conservative estimates of the distribution^75^. Despite these restrictions, existing models provide useful insights into the distribution of suitable habitat and its components under different climate regimes at regional or continental scales. Most fall short of providing a full explanation of how climate change affects the cultural system, however. The explanatory power of SDMs can be improved by developing testable hypotheses that address the question of causality prior to the modelling process, e.g. by introducing relevant variables into the modelling stream and testing them using a variable selection protocol (e.g. refs. ^37,76^).

Contemporary techniques in species distribution modelling help ensure that models are transferable to new environments. For example, proper use of cross-validation leads to more transferability when habitat selection rules can be assumed to be stable^77^ although some species-specific features decrease model transferability, e.g. decreased longevity or strong differences in the response to topographic relief and elevation^78^, these are relevant in multi-species assessments and are not evidence that transferability is not possible, a priori. The largest cross-species comparison of transferability to date^79^, although limited to tree species, provides important lessons applicable to other taxa, including humans. First, although transferability issues may arise regardless of the algorithm used—which suggests that transferability is bounded by the species being predicted—the situation is improved systematically by representing habitat suitability as a quantitative (rather than binary) response. Second, although transferability can decrease when finer-scale environmental layers are coarsened to match the scale of bioclimatic variables^80^, the use of downscaling techniques to increase the resolution of bioclimatic variables, as our workflow suggests, confers protection against low transferability. In what follows, we explore the conceptual gap between SDMs designed, for the most part, to gauge the impact of climate change on the spatial behaviour of past human populations, and models of cultural transmission.

## Building integrated models: a workflow

**Fig. 1:**
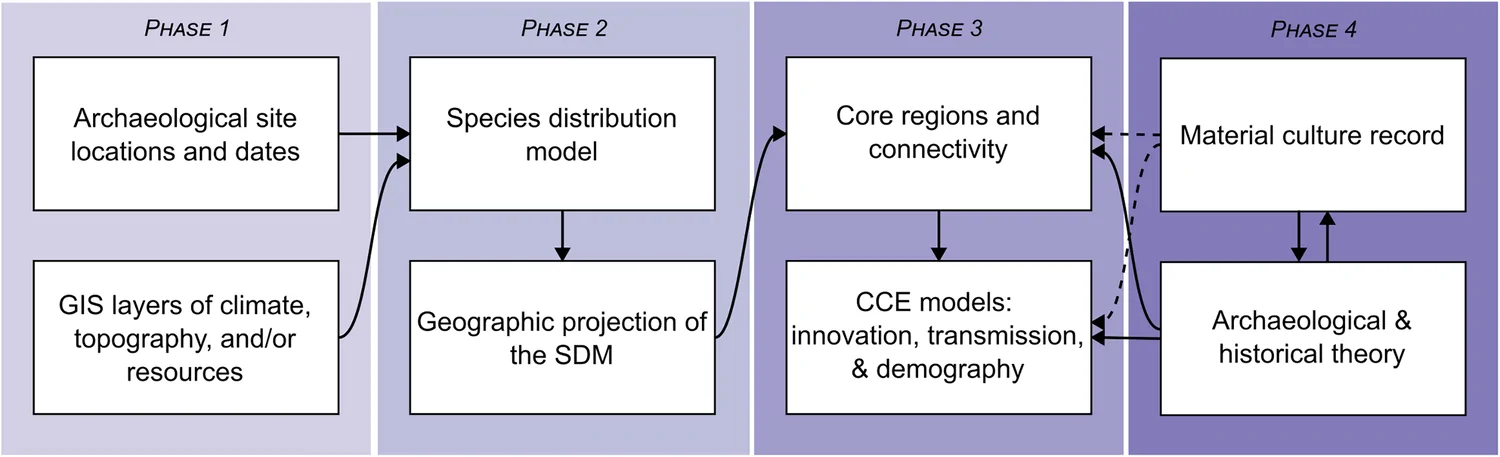
The proposed modelling workflow. Dashed lines indicate use of the archaeological record for model validation.

In this Perspective, we propose an integrative modelling chain (Fig. 1, Box 3) that explicitly uses the spatio-temporal structure of the environment to bridge the gap between climate-informed SDMs and models of cultural evolution. We recommend involving climate scientists from different disciplines (e.g. paleoclimatologists, ecologists and archaeologists) in the model design process to ensure that archaeologically relevant variables are identified prior to running the models to address the issue of causality. Furthermore, by adopting complex systems science as the overall theoretical framework of the analysis, we highlight the importance of fine-grained archaeological analyses of the material culture record and the role of social context and historical contingency in determining the final outcome of human-environment interactions.

The first step in any modelling process is to identify a research question, timeframe and spatial domain for which palaeoenvironmental and archaeological data are available. This step dictates the relevant spatial and temporal scales of the model. Next, a set of environmental variables (e.g. drawn from climate models or digital terrain models) is selected and prepared using a GIS (Fig. 1: phase 1). The preselection of environmental variables should consider the scale of the model and be grounded in theory to ensure that relevant variables are being tested. Climate data with sufficiently comprehensive spatial coverage are usually the products of general circulation models (AOGCMs). Archaeological sites serve as proxies for human presence (the dependent variable) and are selected, controlling for locational and chronological accuracy. One or more modelling techniques (e.g. MaxEnt, Random Forest, Boosted Regression Trees, GLM) are then selected to identify patterns in the distribution of sites relative to environmental variables and make predictions about the relationship between human presence and the physical environment^68^ (Fig. 1: phase 2, top). A stepwise procedure can be used to select relevant variables from the pool of candidate predictors during the modelling process, avoiding some of the misspecification errors mentioned above and providing a means of testing hypotheses^81,82^. Once a numerical model of habitat suitability is produced, it can be projected onto geographic space and visualised (Fig. 1: phase 2, bottom).

In the following step (Fig. 1: phase 3, top), landscape connectivity and ecological network analyses developed in conservation science can be used to quantify the location and size of clusters of suitable habitat and the degree to which they are spatially connected^83,84^. A variety of tools are currently available for implementing these analyses as standalone productions, in R or in GIS environments^85^. Ethnographic and archaeological data can be used to establish the statistical parameters of the models, e.g. the expected range of mobility and the minimum area of core regions, identified using the suitable habitat model, tailoring the analysis to the scale and timeframe of interest. These landscape metrics can, in turn, be used to predict demographic variables such as group size based on habitable area (e.g. refs. ^86,87^) and the density of interactions between human groups using parameters set forth in CCE models (e.g. ref. ^88^).

The next step in the pipeline is to use CCE theory to predict higher-order cultural processes, such as rates of cultural innovation, the probability of transmission of novel traits and their retention at the level of the metapopulation (Fig. 1: phase 3, bottom). Climate change events are linked to changes in habitat structure that influence the demographic structure of human populations, which, in turn, is used to generate testable predictions about the material culture record. There is no circularity because the SDM is generated from locations and environmental variables, whereas the resulting predictions concerning cultural change will be tested using finer-grained aspects of the archaeological record (e.g. assemblage variability)^89^ (Fig. 1: phase 4, top).

Up to this point, the proposed pipeline attributes causality to the adaptive process and is well-suited to predicting climate-driven macroevolutionary change (e.g. speciation and patterns of dispersal), demographic patterns and higher-level processes governed by them (e.g. rates of cultural innovation and cultural transmission). Depending on model design and the criteria used to select variables, culturally relevant hypotheses (e.g. the degree of sensitivity to climate variability) may also be tested. The pipeline is not isotropic and feedback mechanisms between cultural and natural systems (e.g. anthropogenic impacts on vegetation cover^90^) can be incorporated into the modelling process using feedback loops^91^, providing input for the land surface component of GCMs, for example^72^ (Fig. 1: linking phases 3 and 4).

What the proposed pipeline cannot do is predict specific forms of cultural expression (the appearance of given ‘cultural traits’), nor can it explain why a given climate event can have unpredictable outcomes^15^. This level of detail is a product of historical contingency and random, non-linear relationships between the internal (social) and external (natural) variables that constitute the system. Archaeological, ethnographic and historical records provide valuable information about the nature and timing of the fundamental processes that contribute to the emergence of culturally specific properties of the system, e.g. the existence of social inequality, levels of violence, shifting political allegiances, etc. Full integration of human systems into Earth systems models, therefore, requires the compilation of detailed and highly contextualised archaeological and paleoenvironmental information that can be used to disentangle climate change impacts and local-scale cultural processes^3^ and careful consideration of the quality and scale of available data used^7^ (Fig. 1: phase 4).

**Fig. 2:**
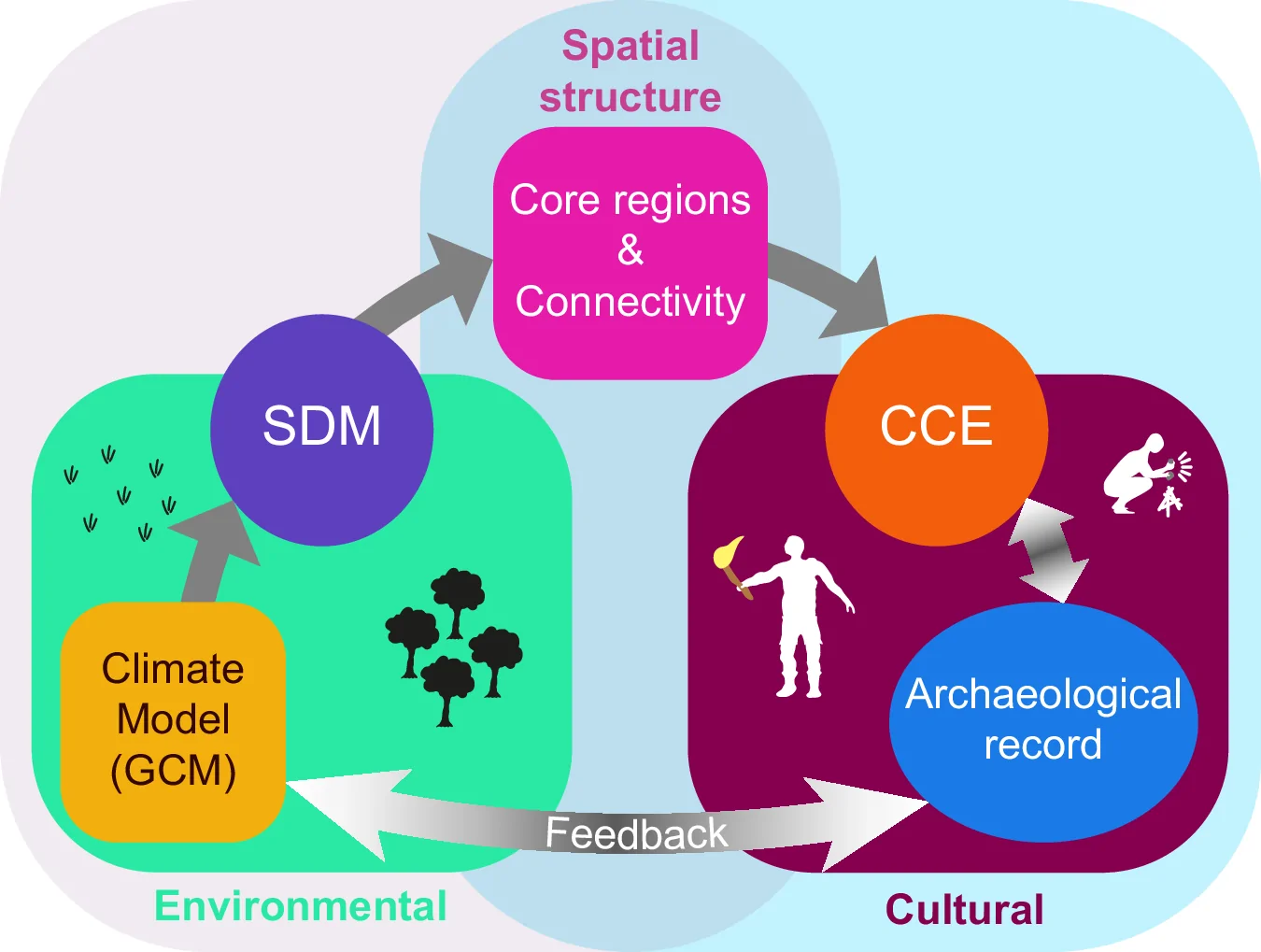
A conceptual illustration of the proposed modelling pipeline. The pipeline is integrative, multiscalar and anisotropic. Climate models and Species distributions models (SDM) produce spatially explicit models of the distribution of human habitat. Cumulative cultural evolution (CCE) links habitat structure and transformations in the cultural system, generating hypotheses that can be tested using the archaeological record and Earth archives.

Finally, it is possible to see how the proposed workflow (Fig. 2) could be expanded and adapted (Box 3). Case studies drawn from the archaeological and palaeontological records (e.g. refs. ^8,92^) generate hypotheses that can be incorporated into initial model design and quantitatively tested. Analogue methodologies involving the use of individual narratives to generate testable hypotheses have been developed by geographers studying the human dimensions of climate change^4^. Agent-based models^93^ can be added to the workflow, e.g. to calculate the probability that a series of small-scale interactions might transform the overall system under specific test conditions.

Box 3 Illustrative case study The proposed workflow, which articulates Earth and cultural systems using the spatial component intrinsic to both (Fig. 1), has yet to be fully completed due to its complex, transdisciplinary nature (see: ‘Building Integrative Models: a Workflow’). Several segments of the workflow are complete, however, and can be combined to illustrate how an Aurignacian case study could be brought to completion. The Aurignacian is an archaeological culture associated with the establishment of modern human populations in Europe roughly 45 to 30 ka.

The first part in the proposed workflow is illustrated in refs. ^133,134^. Phase 1 (Fig. 1: phase 1) consists of selecting an archaeological database and acquiring relevant Earth sciences data for use in the SDM. In our example, this included the creation of a database of securely dated Aurignacian sites with precise geographic coordinates, the acquisition of climate variables representing stadial and interstadial conditions produced by the IPSL CM5A-LR climate model^135^ and a digital terrain model (SRTM 90 m^136^). Data preparation, including the derivation of topographic variables from the digital terrain model and the extraction of predictor variables to a 1 × 1 km grid of presences (Aurignacian sites) and pseudo-absences, was done in a GIS.

The following phase consists of selecting a modelling technique for the SDM (Fig. 1: phase 2). In our example, Random Forest was selected and run iteratively with the dual purpose of selecting a parsimonious suite of predictors and the best SDM for each climate condition, which was then projected onto geographic space. This is the basis for the following step (Fig. 1: phase 3), which consists of identifying regions within the study domain that provide the most suitable habitat and mapping their connectivity. Different approaches exist for this phase (e.g. refs. ^85,137^); in our example, this was accomplished using purpose-built tools in a GIS to produce maps of the distribution of core habitat and hypothetical pathways between them, weighted by the expected frequency with which they would be used^133^. Demographic estimates were not attempted in our case study, but could be produced on the basis of the size of regions and their presumed carrying capacity (e.g. refs. ^87,88^).

The resulting information is now ready for use in a CCE model (Fig. 1: phase 3). CCE makes predictions about rates of cultural innovation, propagation and diversity as a function of parameters that can be derived from the SDM, such as connectivity, population size and risk^138^. In our example, ecological risk is integrated into the SDM modelling workflow and demographic estimates could be derived from the geographic projections of the model (above). The CCE model predictions can then be tested (Fig. 1: phase 4) by applying one of the techniques being developed by archaeologists to analyse evolutionary trends in material culture production (e.g. ref. ^139^). This step should produce valuable information about the influence of climate-driven processes on cultural evolution. It is important to recognise that these processes do not include the social processes that mitigate human responses to the environment and produce local patterns of variability that are not predicted by CCE, however.

The final step is to recognise the limitations of the modelling workflow described so far and consider what empirical approaches and applications of anthropological theory to the interpretation of the archaeological record have to offer (Fig. 1: phase 4). For example, it has been suggested that patterns in the distribution of items of personal adornment in Aurignacian assemblages indicate the existence of ethno-linguistic boundaries^140^, which would potentially affect the patterns of connectivity predicted above (connecting phase 4 to phase 3). This hypothesis could be tested by altering the connectivity field and recalculating the CCE model to see what the impact of the hypothesised social boundaries might have been on the dynamics of climate/culture evolution. Finally, it would be possible to use the SDM to build agent-based models that predict rates of gene flow between the regions (e.g. ref. ^141^), which could then be tested using paleogenetic data and might, in turn, enrich the interpretation of items of personal adornment offered above.

The archaeological record (Fig. 1: phase 4) can be used to test phase 3 model predictions. For example, SDMs can be projected onto novel spatial domains, making predictions about the distribution of suitable habitat that should correlate with site location data in the new domain. Chronological patterns of lithic production can be used to test models representing successive climate cycles, to explore the impact of climate-driven biogeographical barriers, such as the large river valleys of Southwestern Europe^59^.

The proposed workflow builds upon existing scholarship and adopts an integrated, trans- and inter-disciplinary approach to climate science to explore the dynamic interactions between climate, biological, and cultural systems as reflected in the archaeological record.

## Conclusion

In this Perspective, we propose a blueprint for the design of complex models that overcome existing barriers in climate model design and generate predictions about human-climate interactions that are testable using the archaeological record and Earth archives. Climate models and climate change archaeology have been accused of environmental determinism^94–96^ as a result of a historically top-down approach to causality^3,97^ and over-emphasis of the role of readily identifiable and easily quantifiable climate variables as drivers of ecological and social change^15,74^. This potential bias can be overcome by improving the integration of human systems in Earth systems models^5,14,19,23,74^, grounding the modelling process in theory^15^ and generating predictions that can be tested using the archaeological, palaeontological and paleoenvironmental records^8,98^. The proposed pipeline produces an integrated workflow grounded in systems theory that articulates climate-driven SDMs, CCE-derived models of cultural evolution and contextualised, fine-grained analyses of human systems and the material culture record.

In addition to reconciling existing theoretical frameworks, our proposed modelling pipeline is achievable. Environmental and archaeological datasets covering a range of timeframes and geographic locations are now readily available and extensive computing resources, beyond those already in use in most universities and laboratories, are not required. This Perspective, therefore, illustrates how the archaeology of climate science can use existing resources to design fully integrated complex systems models that contribute to climate science and can inform contemporary climate action by providing richly contextualised case studies that demonstrate how past human populations reacted to climate change events and document the outcomes.
